# Role of the microbiota in ileitis of a mouse model of inflammatory bowel disease—Glutathione peroxide isoenzymes 1 and 2‐double knockout mice on a C57BL background

**DOI:** 10.1002/mbo3.1107

**Published:** 2020-08-18

**Authors:** Fong‐Fong Chu, R. Steven Esworthy, Binghui Shen, James H. Doroshow

**Affiliations:** ^1^ Department of Gastroenterology and Hepatology, The First Affiliated Hospital and College of Clinical Medicine of Henan University of Science and Technology Luoyang China; ^2^ Department of Cancer Genetics and Epigenetics Beckman Research Institute City of Hope Duarte CA USA; ^3^ Center for Cancer Research and Division of Cancer Treatment and Diagnosis National Cancer Institute Bethesda MD USA

**Keywords:** IBD, ileitis, *Lactobacillus*, microbiome, mouse model, reactive oxygen species

## Abstract

C57Bl6 (B6) mice devoid of glutathione peroxidases 1 and 2 (Gpx1/2‐DKO) develop ileitis after weaning. We previously showed germ‐free Gpx1/2‐DKO mice of mixed B6.129 background did not develop ileocolitis. Here, we examine the composition of the ileitis provoking microbiota in B6 Gpx1/2‐DKO mice. DNA was isolated from the ileum fecal stream and subjected to high‐throughput sequencing of the V3 and V4 regions of the 16S rRNA gene to determine the abundance of operational taxonomic units (OTUs). We analyzed the role of bacteria by comparing the microbiomes of the DKO and pathology‐free non‐DKO mice. Mice were treated with metronidazole, streptomycin, and vancomycin to alter pathology and correlate the OTU abundances with pathology levels. Principal component analysis based on Jaccard distance of abundance showed 3 distinct outcomes relative to the source Gpx1/2‐DKO microbiome. Association analyses of pathology and abundance of OTUs served to rule out 7–11 of 24 OTUs for involvement in the ileitis. Collections of OTUs were identified that appeared to be linked to ileitis in this animal model and would be classified as commensals. In Gpx1/2‐DKO mice, host oxidant generation from NOX1 and DUOX2 in response to commensals may compromise the ileum epithelial barrier, a role generally ascribed to oxidants generated from mitochondria, NOX2 and endoplasmic reticulum stress in response to presumptive pathogens in IBD. Elevated oxidant levels may contribute to epithelial cell shedding, which is strongly associated with progress toward inflammation in Gpx1/2‐DKO mice and predictive of relapse in IBD by allowing leakage of microbial components into the submucosa.

## Graphical abstract

Hydrogen peroxide (H_2_O_2_) and superoxide are generated by some bacterial genera and by the intestinal mucosa in response to bacteria. Gpx1/2‐double knockout mice are sensitive to intestinal H_2_O_2_ production, which causes crypt apoptosis and anoikis leading to inflammation. We investigate the normal ileum pathology inducing microbiota and use antibiotics to narrow down potential candidates. The principal candidate, *Lactobacillus*, stands out while not demonstrating unambiguous domination in provoking the pathology.

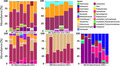

## 1 INTRODUCTION

Wild‐type, transgenic, and gene knockout (KO) rodent models of inflammatory bowel disease (IBD) most often demonstrate dependence on gut microbiota (Kiesler, Fuss, & Strober, 2015; Mizoguchi & Mizoguchi, 2010; Peloquin & Nguyen, 2013). This is in line with discoveries of bacterial alterations in IBD in humans (King & McCole, 2019; Sartor & Wu, 2017; Zuo & Ng, 2018). The question persists whether altered microbiomes cause IBD or are a result of IBD. Rodent models may provide some insight. Identities of provocative microbiota differ and will probably continue to differ model to model (Gkouskou, Deligianni, Tsatsanis, & Eliopoulos, 2014). Suspected pathogens and ordinary commensals might be acting as conditional pathogens depending on the genetic and environmental conditions contributing to each rodent model. Since the genetics of IBD is complex, as are the environments of sufferers, this might be the expectation for humans as well (Liu et al., 2015).

Oxidant generation from enzymes that use reduced nicotinamide adenine dinucleotide phosphate (NADPH oxidases), DUOX2 (H_2_O_2_), and NOX1 (superoxide) and from *Lactobacillus* participate in shaping the gut microbiota (Grasberger et al., 2015; Huang et al., 2016; Jones & Neish, 2017; Knaus, Hertzberger, Pircalabioru, Yousefi, & Branco Dos Santos, 2017; Lipinski et al., 2009; Matziouridou et al., 2018). NOX1‐dependent oxidant generation was observed in C57Bl6 (B6) ilea after oral gavage of *Lactobacillus* but not *Escherichia coli* (Jones et al., 2013). *Lactobacillus* thrives in an oxidant‐rich environment to the detriment of pathogens such as *Citrobacter rodentium and Listeria monocytogenes* (Pircalabioru et al., 2016). This is one basis for proposing the use of *Lactobacillus* sp. as probiotics in the treatment of inflammatory bowel disease (IBD) (Guandalini & Sansotta, 2019). Stimulation of crypt/gland localized NOX1 oxidant generation may be beneficial by sustaining proliferation and restitution (Alam et al., 2014; Coant et al., 2010; Jones et al., 2013; Kato et al., 2016; Singh, Hertzberger, & Knaus, 2018). Intestinal DUOX2 activation is downstream of NOD2 detection of cytosolic muramyl dipeptide, a component of bacteria cell walls (Lipinski et al., 2009). DUOX2 oxidant production may be in response to dysbiosis (Burgueno et al., 2019; Grasberger et al., 2015). Constraints on the expression of host NADPH oxidases and the presence of an antioxidant system normally prevent significant damage from occurring (Chu et al., 2017; Esworthy et al., 2014; Lee, Esworthy, Chu, Pfeifer, & Chu, 2006; Sommer & Backhed, 2015). However, typical oxidant‐producing *Enterococcus faecalis* caused more DNA damage in colon epithelial cells than a mutant strain with attenuated production after oral gavage of a bolus of the bacteria into antibiotic‐pretreated rats (Huycke, Abrams, & Moore, 2002). Oxidant generation in IBD is usually ascribed to ER stress in Paneth cells, NOX2 in macrophages and mitochondria, although *DUOX2* mRNA levels are elevated up to 20 times in active Crohn's ileitis and ulcerative colitis (Hamm et al., 2010; Haberman et al., 2014; Li et al., 2010; Malhotra et al., 2008; MacFie et al., 2014; Yanai et al., 2015).

Glutathione peroxidases (GPX) 1 and 2 are antioxidant isoenzymes that reduce hydroperoxides to water or alcohols (Chu, Doroshow, & Esworthy, 1993). GPX1 is expressed in the luminal epithelium while GPX2 is concentrated in the crypts/glands (Esworthy, Swiderek, Ho, & Chu, 1998). Ileocolitis in homozygous Gpx1/2‐double knockout mice (DKO; *Gpx1*−/−*Gpx2*−/−) occurs spontaneously on 3 backgrounds B6, 129/Sv, and B6.129 (Esworthy et al., 2011; Esworthy, Smith, & Chu, 2010; Lee et al., 2006). Expression of one wild‐type *Gpx1* or *Gpx2* allele (non‐DKO mice; generated as littermates of DKO mice) suppresses the pathology, almost completely on the B6 background (Chu et al., 2017; Esworthy et al., 2014). In B6 DKO ilea, excessive crypt apoptosis and anoikis result in Paneth cell and crypt depletion from 27 to 35 days of age with infiltration of monocytes and neutrophils occurring around 27–28 days of age; pathology reaches its peak around 35 days of age (Chu et al., 2017; Chu, Esworthy, Shen, Gao, & Doroshow, 2019). Colon pathology begins soon after birth.

Studies were performed that link pathology in DKO ilea to DUOX2, NOX1, and microbiota. A germ‐free B6.129 Gpx1/2‐DKO colony did not exhibit pathology (Chu et al., 2004; Esworthy, Binder, Doroshow, & Chu, 2003). Ileocolitis in B6 DKO mice is driven by DUOX2 and NOX1 (Chu et al., 2017; Esworthy et al., 2014). This was shown in triple knockout lines (TKO), one in which the *Nox1* gene was knocked out and the second in which the *Duoxa* locus (*Duoxa1* and *Duoxa2* maturation subunit genes) was modified to eliminate cell surface expression of DUOX1(barely expressed in the intestine) and DUOX2 and the ability to generate H_2_O_2_ (Grasberger et al., 2012). Lack of functional DUOX2 (*Gpx1*−/−*Gpx2*−/−*Duoxa*−/− TKO) eliminated crypt anoikis (exfoliation) and crypt abscesses. Excessive apoptosis remained resulting in partial loss of Paneth cells and crypts and slightly above background levels of macrophage numbers and monocyte infiltration (Chu et al., 2017). *Gpx1*−/−*Gpx*2−/−*Nox1*‐/Y or *Nox1*−/− (*Nox1* gene on X‐chromosome) TKO mice had virtually no pathology (Esworthy et al., 2014).

Our hypothesis for linking microbiota to ileitis was based on the idea that *Lactobacillus*‐induced NOX1 oxidant generation produces damage on its own, which DUOX2 augments. We posited that antibiotic‐induced differences in *Lactobacillus* abundance in the ilea of B6 Gpx1/2‐DKO mice would be reflected in levels of pathology and *E*. *coli* abundance might not show a positive association with pathology since *E*.* coli* did not elicit NOX1 oxidant generation. To examine this, antibiotics were orally administered at the onset of ileum pathology to alter the evolution of the microbiota and possibly influence pathology levels. Three antibiotics were selected to be separately administered and produce distinct outcomes, impacting many operational taxonomic units (OTU; largely genus level). The primary analysis would be based on the association of abundance and pathology marker levels among the antibiotic‐treated and control sets. We were uncertain about the similarity of microbiotas between Gpx1/2‐DKO and non‐DKO mice (*Gpx1*+/−*Gpx*2−/− and *Gpx1*−/−*Gpx*2+/−). Since Gpx1/2‐DKO and non‐DKO mice were sibs or half‐sibs (cage mates), the differences might have been minor. Pathology in Gpx1/2‐DKO mice might alter the abundance of some OTUs from that of non‐DKO mice. This perspective was used to assess the antibiotic effects. Another viewpoint is that ileitis provoking OTUs might have some growth advantage in the pathological milieu over commensals as defined by abundances in wild‐type or other relatively normal mice (non‐DKO cage mates) and suppress the growth of more benign commensals. Thus, comparisons of OTUs with increased abundance in mice with ileitis compared to non‐DKO controls might indicate provoking candidates (Peloquin & Nguyen, 2013; Pircalabioru et al., 2016; Sartor & Wu, 2017). We also analyzed the results from this latter viewpoint.

## 2 MATERIALS AND METHODS

### 2.1 Mice

A genetically segregating breeding scheme (*Gpx1*−/−*Gpx2*−/− male x *Gpx1*+/−*Gpx2*−/− and *Gpx1*−/−*Gpx2*+/− females) provided homozygous Gpx1/2‐DKO mice and non‐DKO mice from most litters (Chu et al., 2017; Esworthy et al., 2014). Breeders were fed LabDiet 5062 (9% fat; LabDiet, St. Louis, MO). Pups were weaned at 21 days of age onto LabDiet 5061 (5% fat). The weight and condition of mice were recorded daily from 8 to 35 days of age. No morbidity was found. The study used homozygous Gpx1/2‐DKO and non‐DKO cage mates. Both males and females were included. The non‐DKO set was composed of 4 males (2 *Gpx1*+/*Gpx2*−/− and 2 *Gpx1*−/−*Gpx2*+/−) and 2 females (*Gpx1*+/*Gpx2*−/−). On the B6 background, there is no discernable difference in ileum histology between *Gpx1*+/1*Gpx2*−/−, *Gpx1*−/−*Gpx2*+/− mice and both genotypes closely resemble wild‐type mouse in histological features (Esworthy et al., 2014). Ileum histology of Gpx1/2‐DKO male and Gpx1/2‐DKO female mice is very similar, different only in the Paneth cells incidence marker.

### 2.2 Antibiotics

Metronidazole, streptomycin, and vancomycin were administered in drinking water; metronidazole concentration was 750 mg/L, streptomycin‐450 mg/L, and vancomycin‐50 mg/L (Ferreira et al., 2011; Sekirov et al., 2008; Wlodarska et al., 2011). Mice shun the metallic taste of metronidazole (Chu, Esworthy, Doroshow, & Shen, 2016). To overcome this, the water used to prepare antibiotics and the water for the control mice contained 0.4% of the sweetener, Splenda®. Mice were allowed free consumption from 22–35 days of age. The treated water was refreshed on the 7th day. At 35 days of age, the mice were euthanized by CO_2_ inhalation. Eighteen to 20 mice were in each group. Mice from multiple litters were used for each set (Splenda‐4 litters; Gpx1/2‐DKO and non‐DKO; metronidazole‐5 litters; vancomycin‐5 litters; streptomycin‐5 litters). The numbers of mice used were adequate to statistically distinguish sets of mice with intermediate pathology scores and marker values from DKO and non‐DKO or wild‐type mice in our previous studies (Chu et al., 2017; Esworthy et al., 2014). Eight Gpx1/2‐DKO mice with overall marker scores representing the average or median and 6 non‐DKO mice were selected for microbiome analysis. The number was based on examining comparable studies in B6 mice where the group sizes were generally 5–9 mice (Garidou et al., 2015; Gu et al., 2013; Jakobsson et al., 2015; Kar et al., 2017; Robertson et al., 2013; Tourret et al., 2017; Walk, Blum, Ewing, Weinstock, & Young, 2010).

### 2.3 Tissue sampling

The length of the small intestine (pylorus to ileocecal junction) was measured and the fecal stream of the ilea, 1–5 cm above the cecum, was expressed into sterile tubes and immediately frozen (−80°C). The length of the colon was measured. Ileum sections were fixed for histology (10% phosphate‐buffered formalin). Samples were processed for sectioning and stained with hematoxylin and eosin (H&E).

### 2.4 Histopathology

**FIGURE 1:**
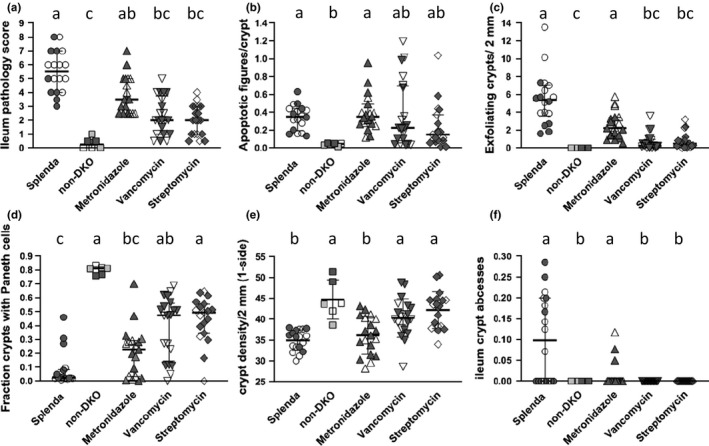
Ileum pathology scores and pathology marker levels in antibiotic‐treated DKO mice, Splenda‐treated DKO mice, and non‐DKO mice. Panel (A) is the overall score derived by adding values from appraisals of apoptosis (apoptosis figures/crypt >0.1 = 1; less = 0), Paneth cells (0.8–1 fraction crypts with Paneth cells = 0; 0–0.79 = 0.5; 0.34–0.59 = 1; 0.15–0.33 = 1.5; 0–0.14 = 2), crypt density (crypts/10× field one side; ≥39.1 = 0; 33.6–39 = 1; 27.8–33.5 = 2), inflammation foci/10× field both sides(0 = 0; 0.01–0.2 = 1; 0.21–0.5 = 2), and inflammation intensity (peak damage in section‐small abscesses = 1; large abscesses = 2; epithelial erosion/ulceration = 3; latter rarely found in B6); median and interquartile range. Panel B shows levels of crypt apoptosis, mean, and standard deviation. Panel (C) shows the fraction of crypts with anoikis (crypt exfoliation), median, and interquartile range. Panel (D) shows the fraction of crypts with Paneth cells, median and interquartile range. Panel (E) shows the number of crypt per 10× field, mean and standard deviation. Panel F shows the number of crypt abscesses per 10× field, median, and interquartile range. Data shown as median and interquartile range were evaluated by the Kruskal–Wallis test for multiple comparisons; data shown as mean and standard deviation were evaluated with 1‐way ANOVA with Tukey's multiple comparisons test. Common letters indicate no significant difference, otherwise a > b, etc. Open symbols for DKO sets (Splenda, metronidazole, vancomycin, and streptomycin) are males, dark symbols are females. For the non‐DKO mice, open symbols are Gpx1+/−Gpx2−/− males, dark symbols are Gpx1+/−Gpx2−/− females and half‐tone symbols are Gpx1−/−Gpx2+/− males

H&E histopathology was evaluated by enumerating crypt apoptosis, crypt exfoliation (anoikis), depletion of crypts and Paneth cells, and crypt abscesses, details in Figure 1 (Chu et al., 2017). The individual scoring was blinded to the identity of the slides.

### 2.5 Isolation of DNA from ileum fecal stream, processing, and analysis of microbiome

DNA was isolated from the ileum fecal stream following the procedure described (Elson, Cong, Qi, Hershberg, & Targan, 2006; Esworthy et al., 2010). DNA was diluted to 0.1 μg/μl in TE (10 mM Tris, 1 mM EDTA, pH 8.0) and submitted to the Integrative Genomics Core City of Hope for processing and analysis. Amplicons of the V3‐V4 16S rRNA region were sequenced using the Illumina MiSeq platform. Duplicate 50‐μl PCRs were performed, each containing 50 ng of purified DNA, 0.2 mM dNTPs, 1.5 mM MgCl2, 1.25 U Platinum Taq DNA polymerase, 2.5 μl of 10× PCR buffer, and 0.5 μM of each primer designed to amplify the V3‐V4: F (5′‐CCTACACGACGCTCTTCCGATCTCCTACGGGNGGCWGCAG‐3′) and R (5′‐GTGACTGGAGTTCAGACGTGTGCTCTTCCGATCTGGACTACHVGGGTWTCTAAT‐3′). Cycling conditions were 94°C for 3 min, then 15 cycles of 94°C for 50 s, 51°C for 40 s, and 72°C for 1 min. 72°C for 5 min is used for the final elongation step. Amplicons were purified using the AxyPrep Mag PCR Clean‐up kit (Thermo Fisher Scientific). Up to 15 ng of PCR products were carried forward to library preparation using second‐round PCR. The Illumina primer PCR PE1.0 and index primers were used to allow the multiplexing of samples. Eight cycles of enrichment PCR were performed, and final libraries cleaned with AxyPrep Mag PCR Clean‐up kit. The library was quantified using ViiA™ 7 Real‐Time PCR System (Life Technologies) according to manufacturer's instructions and visualized for size validation on an Agilent 2100 Bioanalyzer (Agilent Technologies) using a high‐sensitivity DNA assay. The sequencing library pool was diluted to 4 nM until run on a MiSeq desktop sequencer (Illumina). 600 cycles chemistry (Illumina) was used according to the manufacturer's instructions to run the 6 pM library with 20% PhiX (Illumina).

Reads were merged using Mothur (v.1.33.3) *make*.*contigs* function and merged reads with any ambiguous bases were removed using *screen*.*seqs* function. Length selection of the merged reads was performed to retain merged reads with the length between 400 and 470 bp (range of expected V3 and V4 amplicon length), which were then classified against the SILVA ribosomal RNA gene database at the genus level using *classify*.*seqs* function with a confidence cutoff of 80%. Genera (or other OTUs) with abundance greater than 0.5% in at least one sample were included in the principal component analysis (PCA), which was performed based on Jaccard distance of abundance values. Differentially abundant genera or other OTUs in pairwise comparisons were identified requiring a fold‐change of 1.2 at limma‐voom FDR of 0.05. No samples submitted to the core were excluded from the analyses. Abundances and comparison sets (fold differences, *p*‐values, and FDR) are found in supporting data files (Esworthy, Chu, Shen, & Doroshow, 2020): https://doi.org/10.6084/m9.figshare.12167592

Evaluation of the OTUs as pathology candidates was based in part by identifying mice representing the extremes of pathology and analyzing OTUs between the groups for fold differences and statistical significance of the differences (pairwise *t* test and Bonferroni post‐test correction for multiple comparisons). This was followed up by ranking the results of the linear correlation of individual OTU abundance with the pathology scores and marker values from each of the DKO mice. This analysis used all results from Gpx1/2‐DKO mice in the Splenda control and antibiotic treatment groups. A third analysis was based on finding increased OTU abundances in Gpx1/2‐DKO versus non‐DKO mice (aggressive OTUs) or decreased in the abundance of OTUs in Gpx1/2‐DKO mice (protective OTUs). The evaluation of the statistical significance of differences for all analyses are presented in the supporting data: https://doi.org/10.6084/m9.figshare.12167592 [Excel file NONDKO vs. SPLENDACTRL and 13 other files; see Data Availability Statement] (Esworthy et al., 2020).

### 2.6 Statistical analysis

GraphPad Prism 7.01 was used for statistical analysis of pathology. Each data set was a check for a parametric distribution. Parametric sets were analyzed with a *t* test and nonparametric sets with the Mann–Whitney test with post‐tests for multiple samples. Post‐test adjustment for multiple samples was performed for all samples as described in the figure legends.

## 3 RESULTS

### 3.1 Moderately different microbiome in the non‐DKO cage mates

**FIGURE 2:**
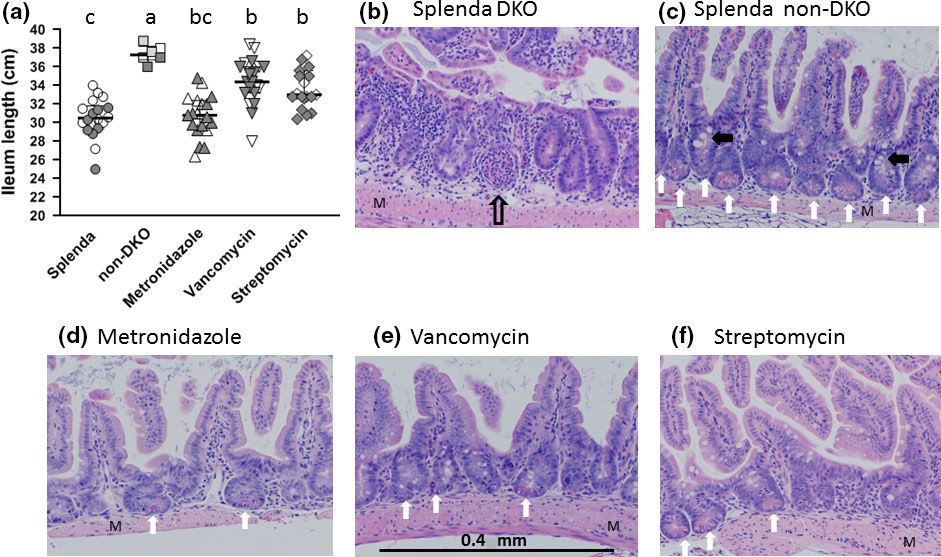
Impact of treatments or genotype on length of the small intestine (mean and standard deviation) panel A. Open symbols for DKO sets (Splenda, Metronidazole, vancomycin, and streptomycin) are males, dark symbols are females. For the non‐DKO mice, open symbols are Gpx1+/−Gpx2−/− males, dark symbols are Gpx1+/−Gpx2−/− females and half‐tone symbols are Gpx1−/−Gpx2+/− males. The values for non‐DKO mice resemble results from wild‐type mice. Statistics 1‐way ANOVA with Tukey's multiple comparisons test. Common letters indicate no significant difference, otherwise a > b, etc. Representative ileum histology (H&E) (panels B‐F). For histology, the original magnification was 10×. Non‐DKO mice (panel C) display histology nearly identical to wild‐type B6 with dense and orderly crypt/villus units (Esworthy et al., 2014). Most crypts have abundant Paneth (vertical white arrows) cells and goblet cells are visible (black arrowhead points to clusters). The control Splenda DKO mice (panel B) have disordered and distorted crypt/villus units that are less densely packed and lack Paneth and goblet cells. This particular image shows a probable crypt abscess (vertical open arrow). Panels D‐F show the generally beneficial impact of antibiotic treatment with some restoration of Paneth and goblet cells in better ordered crypt/villus units. These panels also show that some pathology remains, for example, less densely packed crypt/villus units in the metronidazole set (panel D)

**FIGURE 3:**
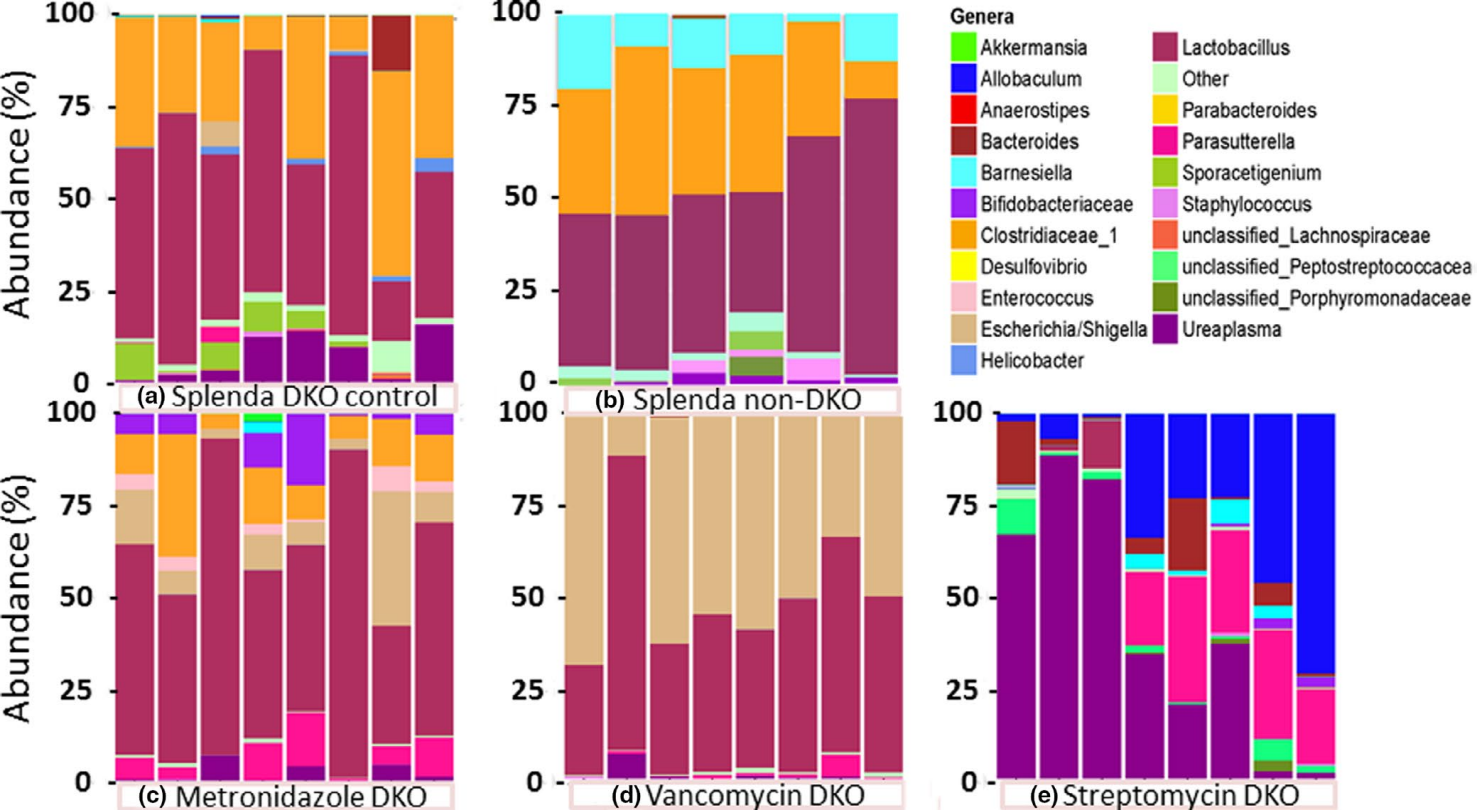
The microbiome of the Splenda control (panel a DKO; Splenda non‐DKO panel b) and antibiotic treated sets (panels c‐e; metronidazole, vancomycin, and streptomycin). Abundances of OTUs of each mouse are shown in a single column as a color spectrum portrayal generally at the genus level; exceptions noted for *Clostridiaceae* for which cluster 1 is shown and unclassified *Lachnospiraceae*, *Peptostreptococcaceae*, and *Porphyromondaceae*, which represent families. Bacteria OTU color codes are shown top to bottom in the same order as the color key—alphabetical). Table 1 provides the mean abundances for each OTU

**FIGURE 4:**
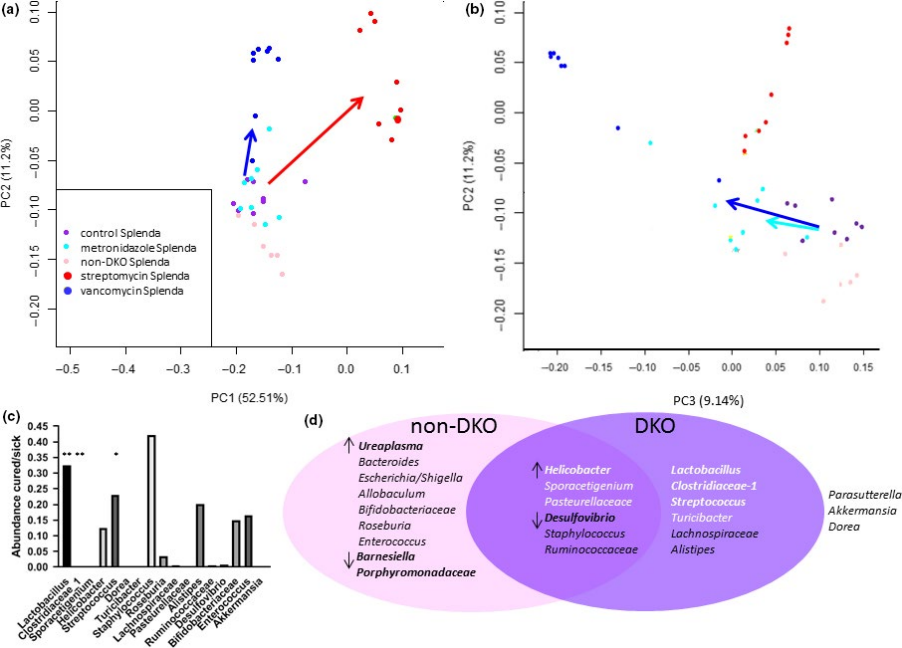
Results of 3 analyses for candidate provocative and beneficial OTUs. Principal component analysis of OTUs showing clustering of mice from Splenda DKO, non‐DKO, and antibiotic‐treated groups. This is based on Jaccard distances using abundance data. Panel a shows PC1 and PC2. Colored arrows indicate diverse microbiome groupings created by antibiotics relative to the Splenda DKO control; dark blue—vancomycin; red—streptomycin. Panel b has results for PC2 and PC3 and shows that non‐DKO mice remained grouped with the Splenda DKO control while the metronidazole group is slightly distanced (light blue arrow). Panel c represents the assessment provocative of OTUs (abundance in low pathology mice ÷sick mice <1). Statistical significance in pairwise *t* tests was found for an abundance of 3 OTUs (* and **). None passed adjustment for multiple samples. The best 2 OTU candidates are indicated by **. Panel d groups candidate OTUs based on contraction in DKO vs non‐DKO (↓) or expansion (↑) (statistically significant differences indicated by bold type), moderate to strong correlation with pathology markers (DKO purple oval stronger candidates in white letters; bold type for best candidates) or both (overlapping pink and purple ovals). The best candidates based on correlation are in bold white. The best consensus candidates are in whites letters and indicated by ↑ in the overlapping region. 3 OTUs found in Table 1 fall outside of both classifications. Data for results shown in panels c and d are found in Supporting Data Files

Pathology scores and small intestine lengths in the Splenda control Gpx1/2‐DKO and non‐DKO mice were consistent with prior observations (Figures 1 and 2). Splenda control Gpx1/2‐DKO ilea harbored microbiomes with some differences from non‐DKO cage mates (Figure 3). In DKO mice, significant decreases in abundance were found for *Barnesiella*, *Desulfovibrio*, and *Porphyromonadaceae* (11–27 fold; 0.005, FDR; Supporting Excel file: NONDKO‐vs‐SPLENDACTRL). *Helicobacter* had a significantly higher abundance in Gpx1/2‐DKO mice (9‐fold; 0.05, FDR) while *Ureaplasma* abundance was nearly 10‐fold higher, falling just short of significance (0.08, FDR). Eight OTUs are greater than 2‐fold more abundant in the Gpx1/2‐DKO mice compared to the non‐DKO mice and 2 other OTUs are more than 2‐fold less in the non‐DKO (not significant) (Figure 4d).

A core microbiota (less than 2‐fold difference; *p* > 0.09) was identified consisting of 8 OTUs accounting for 66.5% of the total in non‐DKO and 73% in DKO mice (*Lactobacillus*, *Clostridiaceae*‐1, *Streptococcus*, *Lachnospiraceae*, *Turicibacter*, *Parasutterella*, *Dorea*, and *Alistipes*). Principal component analysis (PCA), based on Jaccard distance of abundance values, places the Splenda control Gpx1/2‐DKO and non‐DKO sets as close neighbors, partially separated on the PC2 axis (Figure 4a,b).

### 3.2 Antibiotic effect on the ileum microbiome

Streptomycin depleted families *Clostridiaceae*, *Enterobacteriaceae*,* Helicobacteraceae*,* Lactobacillaceae*, and *Pasteurellacease* (Figure 3a,e). *Clostridiaceae* and *Lactobacillaceae* went from the 2 most abundant families (71% combined) to the minor presence (1.9%). The major opportunistic OTUs were *Ureaplasma*,* Allobaculum*, and *Parasutterella*. With metronidazole, *Lactobacillus* abundance was maintained while *Clostridiaceae*‐1 declined to one‐half (not significant; Supporting Excel file: METRO‐vs‐SPLENDACRTL). Of the rest of the most abundant genera, 4 declined in abundance while *Escherichia*/*Shigella* and *Parasutterella* increased in abundance each by 10‐fold. With vancomycin, *Lactobacillus* abundance was maintained while *Clostridiaceace*‐1 was down by 260‐fold. PCA showed partial to complete separation of the antibiotic treatment groups from DKO and non‐DKO mice (Figure 4a,b).

### 3.3 Antibiotic treatment effect on ileitis

A filter for assessing OTUs would be abundances in responsive and unresponsive Gpx1/2‐DKO mice. However, the penetrance of ileum pathology is 95%+; pathology scores were no less than 3 among 53 Gpx1/2‐DKO mice analyzed for this study and in prior work (Figure 1A) (Chu et al., 2019; Esworthy et al., 2014). Mice with scores of 3 exhibit elevated crypt apoptosis with reduction of Paneth cell incidence by half and/or crypt density less than the normal range. Non‐DKO mice had scores from 0 to 1 (Figure 1A). Antibiotic treatment expanded the range of pathology scores in the DKO mice from 0.5 to 8 (Figure 1A). Streptomycin and vancomycin had strong effects on the ileum pathology, while metronidazole had a moderate impact. Even though streptomycin treatment produces almost complete turnover of the microbiome, some pathology remained. Mice representing the extremes from DKO Splenda controls and antibiotic‐treated mice were identified and the OTU abundances compared. Six mice were deemed cured, having pathology scores of 0.5–1 (mean score 0.83 ± 0.26), comparable to non‐DKO mice. Ten mice were selected to represent the sick extreme, having pathology scores of 5 and above (mean score 5.9 ± 0.88). Seventeen OTUs were identified as possibly contributing to pathology by finding values less than 1 for—abundance in cured animals÷abundance in sick animals (Figure 4c). Second, we evaluated statistical significance for the difference in abundance for the respective OTUs (Supporting Excel file: Cured vs. Sick). Based on this, *Lactobacillus*, *Clostridiaceae*‐*1*, and *Streptococcus* stand out as candidate provocative OTUs (*p*‐value ≤0.026, pairwise comparisons). When corrected for multiple samples, the *p*‐value is 0.074 for *Clostridiaceae*‐*1*, 0.088 for *Lactobacillus*, and 0.44 for *Streptococcus*. Seven OTUs show suggestive signs as possible beneficial groups or opportunists by having abundance in cured animals÷abundance in sick animals greater than 1 (*Ureaplasma*, *Bacteroides*, *Escherichia*/*Shigella*, *Parasutterella*, *Barnesiella*, *Allobaculum*, and *Porphyromonadaceae*). The abundances of these OTUs are not significantly elevated in the cured mouse set after post‐test, multiple samples adjustment, although *Bacteroides* and *Ureaplasma* pass the pairwise *t* test (*p* ≤ 0.039).

**FIGURE 5:**
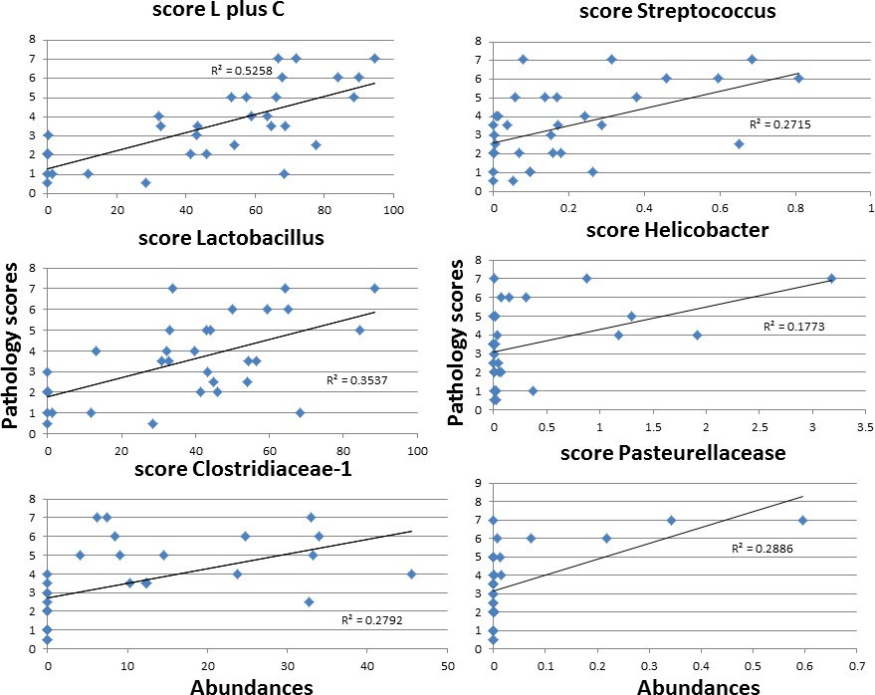
Correlation of OTU abundances with pathology scores using data from antibiotics and Splenda DKO sets. L plus C represents adding the abundances of *Clostridiaceae*‐1 and *Lactobacillus* and performing the correlation versus pathology score. This result was generated for comparison with the *Clostridiaceae*‐1 and *Lactobacillus* correlations. The remaining panels show some of the top candidates based on correlations across all markers (see Table 1 correlations pathology column)

To determine whether any individual OTU might be regarded as primarily responsible for pathology, we tested correlations between OTU abundances and pathology marker parameters, including final score, for all mice from the Gpx1/2‐DKO Splenda control and antibiotic sets. The best results were obtained with *Lactobacillus*, *Clostridaceae*–1, *Pasteurellaceae*, *Streptococcus*, and *Helicobacter* (Table 1a‐pathology column). The coefficients of 7 OTUs are listed with a minus sign indicating an inverse relationship between abundance and pathology; this set is identical to those identified as beneficial above. In many cases, the positive correlation reflects a uniform reduction in abundance in the presence of antibiotics (*Helicobacter*, e.g., Table 1a). *Lactobacillus*, *Clostridaceae*–1, and *Streptococcus* deviate from this pattern. Four OTUs show poor overall correlation with pathology markers, *Dorea*, *Roseburia*, *Bifidobacteriaceae*, and *Enterococcus*. Not all of the positive correlations look robust. Inspection of the correlation graphs reveals full variation in pathology markers values (i.e., pathology scores of 0.5–7) occur in mice with nearly zero abundance of *Helicobacter*, *Pasteurellaceae*, *Sporacetigenium*, and *Turicibacter* (Figure 5 and Figures S2‐S8). The low abundance *of Lactobacillus* and *Clostridaceae*–1 is more consistently associated with lower pathology for markers, while *Streptococcus* shows a mixed pattern. The *Lactobacillus* correlation with pathology score had a modest R‐squared value of 0.35. *Clostridiaceae*‐1 is in competition with *Lactobacillus* based on abundance and strength of correlation (*R*‐squared = 0.28 for pathology score). The *R*‐squared value is increased to 0.53 in the correlation of the combined abundances of *Lactobacillus* and *Clostridiaceae*‐1 to pathology scores (Figure 5 and Figures S2‐S7).

**TABLE 1:** (a) Components of the Splenda control microbiome (bacteria OTUs represent genera, family, or cluster‐based on calls by the SILVA ribosomal RNA gene database) ranked by abundance (mean%; gray highlight)

Bacteria	Abundance					Correlations
Genus/family/cluster	Splenda	Non‐DKO	Metron	Vanco	Strep	Pathology
*Lactobacillus*	44.6	39.54	55.97	43.4	1.77	0.506
*Clostridiaceae‐1*	26.37	25.05	12.8	0.1	0.11	0.455
*Ureaplasma*	6.3	0.64	2.19	0.107	32.38	−0.294
*Sporacetigenium*	3.76	0.68	0.1	0.1	0.1	0.343
*Bacteroides*	1.81	0.45	0.1	0.349	4.4	−0.227
*Helicobacter*	1.22	0.14	0.11	0.11	0.17	0.378
*Escherichia/Shigella*	0.949	0.11	10.62	44.9	0.1	−0.224
*Parasutterella*	0.656	0.46	6.12	1.23	11.73	−0.266
*Streptococcus*	0.507	0.26	0.33	0.1	0.133	0.403
*Turicibacter*	0.477	0.32	0.2	0.1	0.1	0.308
*Dorea*	0.465	0.34	0.1	0.1	0.113	0.088
*Staphylococcus*	0.417	1.34	0.14	0.19	0.159	0.144
*Roseburia*	0.39	0.1	0.1	0.1	0.1	0.085
*Barnesiella*	0.33	8.33	0.348	0.1	1.4	−0.178
*Lachnospiraceae*	0.266	0.27	0.1	0.1	0.104	0.183
*Pasteurellaceae*	0.26	0.1	0.1	0.1	0.1	0.435
*Alistipes*	0.21	0.28	0	0	0.001	0.174
*Ruminococcaceae*	0.19	0.44	0.1	0.1	0.1	0.139
*Allobaculum*	0.16	0.03	0.07	0	20.63	−0.314
*Desulfovibrio*	0.11	1.31	0	0	0.0002	0.175
*Porphyromonadaceae*	0.11	1.17	0	0	0.58	−0.299
*Bifidobacteriaceae*	0.11	0.016	5.86	0	0.79	0.099
*Enterococcus*	0.1	0.01	2.65	0.00065	0.044	0.044
*Akkermansia*	0	0.0003	0.25	0	0	0.122
Total abundance	89.77	82.4	98.36	91.29	75.12	

Pathology parameters					
Pathology parameters	Splenda	Non‐DKO	Metron	Vanco	Strep
Score	5.5	0.25	3.5	2	2
Apoptosis	0.34	0.04	0.39	0.38	0.24
Exfoliation	5.53	0	2.5	0.65	0.77
Paneth cells	0.09	0.8	0.21	0.4	0.44
Crypt density	35	44.7	36.2	40.4	42.2
Abscesses	0.1	0	0.01	0	0

Note

Mean abundances for the non‐DKO mice and antibiotic‐treated mice (metron—metronidazole; vanco—vancomycin; and strep—streptomycin) are listed in adjacent columns. The last set of values (correlations) represent the average of the correlation coefficients for the pathology parameters listed in (b) for each mouse's pathology parameters versus the abundance of the OTUs for each mouse; all coefficients converted to positive values when larger OTU abundance is associated with greater pathology and converted to all negative values when larger OTU abundance is associated with less pathology. In (b), the median pathology score, the median level of crypt exfoliation, the median of the fraction of crypts with Paneth cells, the median value for crypt abscesses, and mean value for crypt apoptosis are listed.

## 4 DISCUSSION

We examined nine publications to determine whether the microbiomes found in the Gpx1/2‐DKO and non‐DKO ilea were typical of B6 mice (Garidou et al., 2015; Gu et al., 2013; Jakobsson et al., 2015; Kar et al., 2017; Mastrocola et al., 2018; Matziouridou et al., 2018; Robertson et al., 2013; Tourret et al., 2017; Walk et al., 2010). The reported microbiomes differed extensively, in some cases between replicate experiments within studies. One paper did not test for *Clostridiaceae*‐1 or a larger taxonomic unit containing the cluster and in others, *Clostridia*/*Clostridium* designation indicates clusters XIVa and IV. *Lactobacillaceae* or *Lactobacillus* abundances averaged 18% ± 21. *Clostridia*/*Clostridium* represented 5% ± 6 of reads with mice on 5001 and 5021 diets and a semi‐synthetic diet at the higher end of the range‐5%, 5%, 11%, and 18% (*Clostridium* sensu stricto, *Clostridiaceae*, *Clostridia*, and *Clostridium*) (Jakobsson et al., 2015; Mastrocola et al., 2018; Tourret et al., 2017; Walk et al., 2010). *Clostridium* sensu stricto corresponds to *Clostridiaceae*‐1 (Gupta & Gao, 2009). The bulk of reads among the sets consisted of *Turicibacter* (50%), unclassified‐*Bacteriodales* (38%), S24‐7 (2 reports; 63% and 55%), MIB (40%), *Porphyromonadaceae* (65%), *Erysipelotrichaceace* (2 reports; 22% and 50%), *Lachnospiraceae* (35%), and *Allobaculum* (22%). Our results may be one more example of the diversity among B6 ilea microbiomes, the high co‐abundance of *Lactobacillus*, and *Clostridiaceae*‐1 representing a unique combination. A link between facultative anaerobes like *Lactobacillus* and anaerobic *Clostridia* has been suggested to be due to the consumption of oxygen by *Lactobacillus* establishing anaerobic niches for *Clostridia* to thrive (Laukens, Brinkman, Raes, De Vos, & Vandenabeele, 2016). Not mentioned in reports on laboratory B6 mice but found in wild mice is *Mycoplasma* (*Ureaplasma*), found in the ilea of our mice (Suzuki & Nachman, 2016). *Ureaplasma's* status as a pathogen is unclear in humans (Combaz‐Sohnchen & Kuhn, 2017). *Ureaplasma* is a candidate as a provoking OTU based solely on the Gpx1/2‐DKO vs. non‐DKO comparison. Based on the other analyses *Ureaplasma* does not appear to be a pathogen in Gpx1/2‐DKO mice and is categorized as a potentially beneficial OTU, or as likely, an opportunistic OTU. *Helicobacter* was accidentally introduced into B6.129 Gpx1/2‐DKO mice soon after the line was established and transmitted to the B6 line (Esworthy et al., 2001). *Helicobacter* has long been noted as a possible pathogen in many rodent models of IBD (Peloquin & Nguyen, 2013). *Helicobacter* is the only OTU to show up as a tentative candidate in all 3 analytical approaches. However, it emerges as a strong candidate only in the Gpx1/2‐DKO vs. non‐DKO comparison with greater abundance in the Gpx1/2‐DKO.

A second question is whether the antibiotic treatments produced predictable effects on the microbiome. Our goal in using these 3 antibiotics was to achieve 3 distinct outcomes, which appeared likely based on results from prior studies using wild‐type B6 mice (Ferreira et al., 2011; Ju et al., 2017; Sekirov et al., 2008; Wlodarska et al., 2011). Once the composition of control DKO/non‐DKO microbiomes was determined, we were in a position to retrospectively examine whether the impact of the antibiotics would be expected. Metronidazole is supposed to target anaerobes. Thus, *Clostridiaceae*‐1 abundance should be down. In B6 colon, treatment with metronidazole at the dose used here for 4 days depleted *Clostridium coccoides* (cluster XIVa) and suppressed *Bacteroidales* allowing for increased abundance of *Lactobacilli*, *Bifidobacteriaceae*, and *Enteroccaceae* (Ju et al., 2017; Wlodarska et al., 2011). This is similar to what we found, although the impact on *Clostridia* was not nearly as strong in our samples. Vancomycin depleted Gram‐positive *Clostridiaceae*‐1, as expected. It did not affect Gram‐positive *Lactobacillus*, which could be anticipated due to similar findings in B6 colon at this concentration (Sekirov et al., 2008). The increase in abundance of the Gram‐negative *Escherichia*/*Shigella* OTU might have been expected from the loss of *Clostridiaceae*‐1 or other OTUs, although the overgrowth far exceeded that found in B6 colon at this concentration (Sekirov et al., 2008). The effect of streptomycin in the reference papers was to deplete *C*. *coccoides* and suppress *Lactobacilli* while leaving *Bacteroidales* unaffected. This is what we found in our samples. The only possible anomalies were the partial impact of metronidazole on *Clostridiaceae*‐1 abundance and the level of overgrowth by the *Escherichia*/*Shigella* OTU with a low concentration of vancomycin. The latter finding might be related to the ileum having higher oxygen content than colon favoring more overgrowth by facultative anaerobes like the *Escherichia*/*Shigella* OTU (Sommer & Backhed, 2016).

The Gpx1/2‐DKO and non‐DKO comparison can be used to examine the issue of whether pathology alters the microbiome. Ileum pathology was mild in the newly weaned B6 Gpx1/2‐DKO mice, confined to sporadic crypt exfoliation and sporadic crypt apoptosis that, in the majority of cases, was not over normal levels (Chu et al., 2017, 2019). *Nox1* and *TNF*‐*α* mRNA levels were not elevated at this time. With that context, sibship of Gpx1/2‐DKO and non‐DKO mice and fostering by non‐DKO dams, we would expect they shared very similar microbiotas at weaning. The 2 sets of microbiomes are comparable when pathology reaches its high point (35 days and thereafter), although the non‐DKO microbiome is only marginally less different from the Splenda Gpx1/2‐DKO set than the metronidazole set based on PCA (non‐DKO separates in PC2; metronidazole in PC3). Four OTUs show up as having significantly altered abundances between Gpx1/2‐DKO and non‐DKO while 14 are altered between Gpx1/2‐DKO mice on Splenda and metronidazole‐treated mice (Esworthy et al., 2020). The possible impact of pathology in Gpx1/2‐DKO mice on the microbiota can be used as a reference for evaluating the effects of vancomycin and streptomycin.

Our experiment was designed so that antibiotics would have an opportunity to modify the microbiota before pathology significantly altered the architecture of the ileum with ensuing immune cell infiltration. Based on prior studies, there was a window from 22 to 26 days of age for the antibiotics to operate. Without antibiotics, pathology becomes more aggressive on and after 27 days of age (Chu et al., 2017, 2019). In our view, the early modification of the evolving microbiota would then influence subsequent pathology. The comparison of Gpx1/2‐DKO and non‐DKO microbiomes shows how pathology impacts the microbiota. The general effect of the antibiotics, vancomycin, and streptomycin was to significantly reduce pathology. Both produced a huge difference in the resulting microbiomes from the non‐DKO mice (Figure 4a,b). The antibiotic effect appears to dominate over any differences produced by differences in pathology levels. We seem to be largely observing the impact of microbiota on pathology.

The stratification of mice from the Splenda control and antibiotic Gpx1/2‐DKO sets into cured and sick divides the OTUs into provisional provocative and beneficial or opportunistic categories with statistical analysis for significance in either category. *Bacteroides* and *Ureaplasma* from the beneficial set pass statistical testing for differences in abundance in a pairwise *t* test and fail in after adjustment for multiple comparisons. The correlations with pathology markers for the beneficial set are generally weaker than in the provocative set (Table 1a). *Lactobacillus*, *Clostridiaceae*‐1, and *Streptococcus* were identified as candidate provocative OTUs in this analysis in pairwise testing. All fail to pass after adjustments for multiple samples with *Lactobacillus* and *Clostridiaceae*‐1 remaining as provisional candidates. *Streptococcus* is regarded as a probiotic (Koretz, 2018; Shiina et al., 2015). The literature on *Clostridiaceae*‐1 (*C*.* perfringens* excluded) is generally unfavorable to candidacy as a pathogen (Kanai, Mikami, & Hayashi, 2015; Lawson, Citron, Tyrrell, & Finegold, 2016; Peloquin & Nguyen, 2013; Peyrin‐Biroulet et al., 2012). While some species of *Lactobacillus* are used as probiotics, members of the genera exhibit properties that fit into our notion of a pathogen for Gpx1/2‐DKO mice by eliciting oxidant generation and/or generating oxidants (Jones et al., 2013; Knaus et al., 2017). *Escherichia coli* did not elicit ileum oxidant generation in earlier studies and this analysis, the associated OTU appeared to be eliminated as a provocative candidate. Some species of *Streptococcus* have a pyruvate oxidase activity and under aerobic conditions can generate H_2_O_2_ (Redanz et al., 2018). There is no information on whether *Streptococcus* elicits NOX1 oxidant generation by the host. The abundance is so low that its possible impact by these mechanisms would be minor compared to *Lactobacillus*.

*Lactobacillus* does not show a singular dominance in the correlation analysis with pathology markers and no other compelling candidates emerge. The reason for this may be that we are looking at genera and families rather than species. Alternatively, a superior correlation is found by using the combined abundance of *Lactobacillus* and *Clostridiaceae*‐1 and pathology markers. This improves the average of the correlation coefficients to 0.62 and more consistently associates high pathology maker values with high abundance. We examined this to investigate the idea that the response of Gpx1/2‐DKO mice may be to components of several pathology provoking OTUs. The residual pathology in the streptomycin set indicates at least a low‐level reaction to a different set of bacteria than found in the Gpx1/2‐DKO Splenda control (pathology scores of 3, 2, 2 and 2 for 4 of the last 5 mice in the streptomycin set, Figure 3e).

A second notion is that the displacement of *Clostridiaceae*‐1 by the *Escherichia*/*Shigella* OTU in the metronidazole and vancomycin sets might produce direct competition with *Lactobacillus* for microaerobic niches (Espey, 2013; Sommer & Backhed, 2016). Studies suggest that this would normally be an unlikely circumstance made possible by sustained antibiotic treatment. H_2_O_2_ and lactic acid production by *Lactobacillus* can provide an edge over *E*.* coli* for the population of mucosal sites (Gupta et al., 1998). The impact of high abundances of *E*.* coli* could be a reduction in oxidants both from bacteria and host (Jones et al., 2013). Adjusting for the presence of *Escherichia*/*Shigella* OTU as a factor diluting *Lactobacillus* improves the average of the correlation coefficients to 0.58 (EF supporting file *Lactobacillus* adjusted for *E. coli* dilution and Figure S10) (Esworthy et al., 2020). Catalase, present in many strains of *E*.* coli*, can be a significant sink for H_2_O_2_ (Rodriguez, Peiroten, Landete, Medina, & Arques, 2015). This would be another mechanism by which the *Escherichia*/*Shigella* OTU could counter *Lactobacillus* and host oxidant generation (Rodriguez et al., 2015). While 2 attempts to factor in the interaction between OTUs produce a better fit in correlations with pathology markers, neither yield a spectacularly enhanced outcome and employ opposed models (pathology enhancing interaction vs. counter pathological interaction) to yield similar improvements.

From the collective work on the Gpx1/2‐DKO mouse model (B6, 129/Sv and B6.129 backgrounds), we surmise that ileum and colon cell loss (the latter a major feature on the 129/Sv background) is driven by a subset of the microbiota primarily by stimulation of host oxidant generation (NOX1, DUOX2, and possibly ER stress in Paneth cells) (Chu et al., 2017, 2019). Inflammation is probably a result of leakage of microbial components into the submucosa since there was no indication of sterile inflammation in germ‐free B6.129 Gpx1/2‐DKO mice. In B6 and 129 Gpx1/2‐DKO mice, elevated lipopolysaccharides levels were detected in plasma indicating leakage through the epithelial barrier (Esworthy et al., 2011; Gao et al., 2010). Several mouse IBD models feature ileitis (Samp/YitFc, TNF^∆ARE^, Caspase‐8^∆iec,^ Xbp‐1^∆iec^, NEMO^tamIEC−KO^, FADD^∆iec^, and N‐cadherin∆) (Cominelli, Arseneau, Rodriguez‐Palacios, & Pizarro, 2017). In common with most of these models is distress in or loss of Paneth cells due to aberrant autophagy and ER stress (Adolph et al., 2013). We found that the population of apoptosed and exfoliated cells while including Paneth cells was predominantly other cell types based on the general absence of lysozyme (Chu et al., 2017). Some of these ileitis models (Samp/YitFc, Caspase‐8^∆iec,^ and FADD^∆iec^) exhibit sterile inflammation which can be augmented by the presence of microbes. In models with sterile inflammation, the Paneth cells may undergo necrosis or necroptosis, which are inherently inflammatory (Stolzer et al., 2020). Inflammatory signals may also derive from danger‐associated molecular patterns (DAMPs) generated from defective autophagy and unfolded proteins (unfolded protein response; UPR) in Paneth cells with sub‐lethal distress (Cadwell et al., 2008). Findings from genome‐wide association studies link genes in the UPR and autophagy pathways to Crohn's ileitis and Paneth cell defects (Cadwell, Stappenbeck, & Virgin, 2009). We see an outright loss of Paneth cells in Gpx1/2‐DKO mice, which has been proposed by others to be a possible consequence of ER stress (Kaser et al., 2008). ER stress increases the production of H_2_O_2_ as a by‐product of augmented protein folding activity (Delaunay‐Moisan & Appenzeller‐Herzog, 2015). Indications of ER stress were detected in tissues of B6 and 129 Gpx1/2‐DKO mice at very high levels of pathology and not at moderate levels of pathology (Esworthy et al., 2011; Gao et al., 2010). Since pathology in B6 Gpx1/2‐DKO mice shows a dependence on NOX1, we doubt that ER stress generates the ileitis, although when pathology is underway ER stress may contribute to the Paneth cell loss.

Pathology in the Xbp‐1^∆iec^ and TNF^∆ARE^ models is dependent on microbiota (Adolph et al., 2013; Schaubeck et al, 2016). In the Xbp‐1^∆iec^ model (ER stress‐driven ileitis; B6; 129; FVB mixed background), the demonstration was limited to evaluation of germ‐free mice. As in the Xbp‐1^∆iec^ model, we found nearly complete loss of Paneth cells and only partial loss of goblet cells (Chu et al., 2017; Kaser et al., 2008). In the TNF^∆ARE^ model (TNFα over‐expression driven ileitis; B6/N background), the ilea showed a range of Paneth cell loss with the worst mice being similarly devoid of Paneth cells as Gpx1/2‐DKO mice. When the TNF^∆ARE^ mice were treated with a combination of metronidazole and vancomycin for 4 weeks there was a partial but significant reduction in ileum pathology. That is what we would predict for a combined metronidazole and vancomycin effect on B6 Gpx1/2‐DKO mice. The cecum microbiome analysis showed a positive correlation between the abundance of unknown *Clostriadales* and ileitis scores (*r* = 0.48), while there was a strong negative correlation with *Porphyromonadaceae* (*r* = −0.79). We found a negative correlation for *Porphyromonadaceae* (*r* = −0.32). Unfortunately, we have no basis for comparing unknown *Clostriadales* to *Clostridiaceae*‐1. We are also comparing cecum to the ileum and the abundance of unknown *Clostriadales* was in the range of 1%–4% of total reads as opposed to 25% for *Clostridiaceae* in our samples. TNF^∆ARE^ mice with low ileitis scores had microbiota compositions resembling wild‐type mice; the Splenda control Gpx1/2‐DKO mice closely resembled the non‐DKO controls. Loss of up to one‐half of the Paneth cells in B6 Gpx1/2‐DKO mice, largely through apoptosis, was associated with very mild, sporadic inflammation in Gpx1/2‐Duoxa‐TKO mice at 35 days at age and Gpx1/2‐DKO mice at 29 days at age (Chu et al., 2017). Macrophage numbers were elevated with few infiltrating monocytes and rare crypt abscesses. Further loss of Paneth cells was driven by anoikis/exfoliation in Gpx1/2‐DKO mice. This appeared at a later time than apoptosis and never attained high levels in Gpx1/2‐Duoxa‐TKO mice. While Paneth cell loss B6 Gpx1/2‐DKO mice is nearly complete by 32 days of age, we viewed this loss as a marker for the underlying processes of apoptosis and exfoliation and not a singular cause of inflammation. Lack of Paneth cells in B6 Gpx1/2‐DKO mice was not associated with a marked dysbiosis as gaged by the non‐DKO microbiome. However, increased susceptibility to the core microbiome could be due to the failure of anti‐bacterial defenses normally provided by Paneth cells. Exfoliation of any epithelial cell type can be inflammatory due to leakage of microbial components into the submucosa during this protracted process (Williams et al, 2015). After the treatment of IBD, high levels of exfoliation are prognostic of the potential for relapse (Kiesslich et al, 2012; Turcotte et al, 2012). In this way, we can link a process observed in Gpx1/2‐DKO mice to relapse in IBD and dependence on the microbiota.

## CONFLICT OF INTERESTS

None declared. The content is solely the responsibility of the authors and does not necessarily represent the official views of NIH.

## AUTHOR CONTRIBUTIONS

**R. Steven Esworthy:** Conceptualization (lead); formal analysis (lead); investigation (lead); methodology (lead); visualization (lead); writing – original draft (lead); writing – review & editing (lead). **Fong‐Fong Chu:** Funding acquisition (supporting); writing – review & editing (supporting). **Binghui Shen:** Funding acquisition (lead). **James H. Doroshow:** Funding acquisition (lead).

## ETHICS STATEMENT

Studies were approved by the City of Hope IACUC (protocol #92008), which conforms to NIH and the American Association for Accreditation of Laboratory Animal Care (AAALAC) rules and standards.

## Data Availability

Supporting data sets generated and/or analyzed during the current study are available at figshare (Esworthy et al., 2020): https://doi.org/10.6084/m9.figshare.12167592; Excel files (EF) and supporting figures: (1) EF Abundances of OTUs; (2) EF NONDKO‐vs‐SPLENDACTRL‐differential‐abundance‐stats; (3) EF METRO‐vs‐SPLENDACTRL‐differential‐abundance‐stats; (4) EF STREP‐vs‐SPLENDACRTL‐differential‐abundance‐stats; (5) EF VANCO‐vs‐SPLENDACRTL‐ differential‐abundance‐stats; (6) EF Cured vs sick; (7) EF cured vs non‐DKO, non‐DKO vs metronidazole; (8) EF abundances correl abscesses; (9) EF abundances correl apoptosis; (10) EF abundances correl crypt density; (11) EF abundances correl exfoliation; (12) EF abundances correl Paneth cells; (13) EF abundances correl pathology scores; (14) EF Lactobacillus adjusted for *E. coli* dilution; (15) Figures S1‐S8—correlation graphs of top candidate provocative OTUs abundance with pathology markers; (16) Figure S9—pathology in colon; (17) Figure S10—correlation graphs Lactobacillus adjusted for* E. coli* dilution.
